# Intracardiac Echocardiography‐Guided Radiofrequency Ablation in Atrial Fibrillation

**DOI:** 10.1111/echo.70168

**Published:** 2025-05-28

**Authors:** Jiaqi Wang, Lang Wang, John T. W. Yeow

**Affiliations:** ^1^ Systems Design Engineering University of Waterloo Waterloo Canada

1

Atrial fibrillation (AF) is the most prevalent form of arrhythmia, contributing significantly to cardiac morbidity and mortality [1]. Its clinical manifestations vary among individuals, encompassing palpitations, dizziness, syncope, fatigue, dyspnea, and chest pain, while some patients, particularly in early or intermittent stages, may remain asymptomatic. The management of AF primarily involves pharmacological [2] and non‐pharmacological [3] strategies. Pharmacological treatments focus on restoring and maintaining normal sinus rhythm through antiarrhythmic drugs, alongside anticoagulant therapy to mitigate the risk of thromboembolism and stroke. However, in cases where pharmacological approaches prove inadequate, non‐pharmacological interventions become necessary. These include electrical cardioversion, which employs controlled electric shocks to re‐establish sinus rhythm, and catheter ablation, a procedure that targets aberrant electrical activity within the heart. The choice of treatment is dictated by factors such as AF type, duration, symptom severity, and overall patient health. Among non‐pharmacological options, catheter ablation has emerged as a particularly effective approach, especially for patients refractory to drug therapy [4]. This technique involves delivering radiofrequency or cryothermal energy through a catheter to specific myocardial regions, thereby disrupting or isolating the abnormal electrical pathways responsible for AF. The success of catheter ablation relies on precise localization of arrhythmogenic foci, necessitating the use of advanced imaging modalities. Commonly employed techniques include x‐ray fluoroscopy [5], transesophageal echocardiography (TEE) [6], and intracardiac echocardiography (ICE) [7], each offering distinct advantages in guiding catheter placement and ensuring procedural efficacy.

The significance of the target paper lies in its evaluation of the safety and efficacy of intracardiac echocardiography (ICE)‐guided radiofrequency catheter ablation for treating atrial fibrillation [8]. ICE is an advanced imaging technique that enables real‐time visualization of the heart's internal structures. By positioning an ultrasound probe directly within the heart or in nearby blood vessels, ICE provides high‐resolution images and Doppler capabilities, allowing for detailed assessment of cardiac anatomy and blood flow. The paper highlights the advantages of ICE guidance by comparing it with conventional x‐ray‐guided ablation methods, demonstrating its potential to enhance procedural accuracy and clinical outcomes.

There were 184 patients in the study, with 30 undergoing ICE‐guided ablation (ICE group) and the remaining 154 receiving conventional x‐ray‐guided ablation (non‐ICE group). The study primarily aimed to compare the two groups in terms of procedural efficiency, radiation exposure, complication rates, and post‐procedure medication use. The key findings indicated that the ICE group had significantly shorter septal puncture time, left atrial modeling time, and total ablation duration, suggesting improved procedural efficiency with ICE guidance. Additionally, the ICE group experienced significantly lower radiation exposure, an important factor for both patient and healthcare provider safety. Postoperative complications, including nausea, vomiting, and hypotension, were also less frequent in the ICE group, indicating a superior safety profile. Regarding post‐procedure medication use, the ICE group had a lower incidence of amiodarone use within three months of discharge, suggesting enhanced efficacy in maintaining sinus rhythm. Although both groups exhibited similar 1‐year success rates (80.00% in the ICE group vs. 77.92% in the non‐ICE group), ICE‐guided ablation demonstrated superior short‐term outcomes.

This study represents the first systematic comparison of the clinical outcomes between ICE‐guided and conventional x‐ray‐guided catheter ablation for atrial fibrillation, offering empirical evidence on the advantages of ICE in enhancing procedural efficiency, minimizing radiation exposure, and reducing post‐procedural complications. These findings carry significant clinical implications, highlighting ICE as a potential approach to improving treatment outcomes and accelerating patient recovery. Additionally, the study suggests that ICE‐guided ablation may reduce the reliance on post‐procedural antiarrhythmic medications, providing a novel perspective for future research in cardiac electrophysiology. However, despite these promising results, limitations such as a small sample size and the absence of long‐term follow‐up warrant caution in interpretation. To strengthen these findings and assess their broader applicability, the authors advocate for a multicenter, randomized controlled trial to further investigate ICE's impact on patient prognosis and long‐term quality of life.

While this study provides valuable insights, some limitations exist. A limitation to consider is the relatively small sample size used in this study, with only 184 patients included, of whom just 30 underwent ICE‐guided ablation. The sample size could have influenced the statistical power, and consequently, the reliability and generalizability of the findings. Additionally, the small cohort size likely constrained the ability to perform subgroup analyses, making it difficult to assess the applicability of ICE‐guided ablation across diverse patient populations with varying clinical characteristics. Another notable limitation is the lack of extended postoperative follow‐up. Although patient outcomes were tracked for up to a year, this study emphasized short‐term clinical efficacy and procedural safety. While the study provides insights into short‐term outcomes, the lack of long‐term follow‐up data limits conclusions about the sustained effectiveness of ICE‐guided ablation and its long‐term cardiac impact. Furthermore, the study does not fully account for individual patient differences, such as variations in AF type, disease duration, or coexisting conditions, which could significantly influence ablation outcomes. Failure to consider these factors may introduce bias, affecting the precision of clinical recommendations. Despite these limitations, the study provides compelling evidence supporting the advantages of ICE‐guided AF ablation, underscoring its potential to enhance procedural efficiency and improve patient safety.

This study provides critical evidence supporting the clinical value of intracardiac echocardiography (ICE)‐guided atrial fibrillation (AF) ablation, demonstrating its significant advantages in enhancing procedural efficiency, minimizing radiation exposure, and reducing postoperative complications. Despite these benefits, several limitations remain that must be addressed to fully optimize the clinical application of ICE in AF ablation. The study's limitations—including the reliance on x‐ray guidance, influence of operator experience, limited ICE technology accessibility, small sample size, and lack of long‐term follow‐up—should be considered. To further advance this field, future research should focus on refining the integration of ICE in AF ablation and expanding its applicability across diverse patient populations and clinical environments. A key area for future investigation is the evaluation of ICE‐guided ablation in specific patient subgroups. The current study primarily focuses on general AF patients, without accounting for variations in pathophysiological characteristics among different patient populations [9]. Given that AF manifests with significant heterogeneity, further studies should explore the safety and efficacy of ICE‐guided ablation in elderly patients, individuals with structural heart disease, chronic kidney disease, or obesity. These conditions may influence atrial remodeling, affect procedural complexity, and alter treatment outcomes. A tailored approach to ICE‐guided ablation based on patient‐specific factors will be crucial in refining clinical decision‐making and improving individualized treatment strategies. Another critical direction is the integration of artificial intelligence (AI) to enhance ICE image analysis and catheter navigation. Operator dependence remains a major limitation of ICE‐guided ablation, as procedural success often hinges on physician expertise. AI‐driven advancements have the potential to improve the accuracy and consistency of ICE image interpretation [10], enabling automated detection of cardiac structures, real‐time hemodynamic assessment, and precise localization of ablation targets. The development of deep learning‐based ICE image segmentation and analysis models could significantly enhance the identification of atrial anatomy and lesion sites, thereby reducing procedural variability and improving overall efficiency. Additionally, combining AI algorithms with ICE imaging may facilitate real‐time predictions of optimal ablation sites, improving lesion placement accuracy and increasing long‐term procedural success rates. While ICE guidance in this study demonstrated a reduction in radiation exposure compared to conventional x‐ray‐guided ablation, future research should aim to completely eliminate x‐ray dependence. Currently, ICE is primarily used for transseptal puncture, but x‐ray imaging is still employed in other procedural steps. The transition to a fully x‐ray‐free ablation approach would be particularly beneficial for vulnerable populations, such as pregnant patients or young individuals requiring AF treatment. Enhancing ICE imaging capabilities through AI‐assisted reconstruction techniques could further improve procedural visualization, ultimately replacing fluoroscopy in all stages of catheter ablation. Beyond technological advancements, the widespread adoption of ICE in clinical practice requires addressing practical barriers such as cost, accessibility, and physician training. ICE systems remain expensive, limiting their use primarily to specialized centers, while a lack of standardized training protocols may hinder broader clinical adoption. Future research should explore strategies to reduce equipment costs, streamline physician training programs, and improve proficiency among operators, particularly in primary care hospitals. Expanding the use of ICE to lower‐tier healthcare facilities could enhance treatment accessibility and improve patient outcomes across diverse clinical settings. By addressing these limitations and advancing ICE‐guided ablation through targeted research efforts, future studies can further refine its role in AF management, ultimately optimizing patient care and procedural success. Finally attempts should also be made to integrate capacitive micromachined ultrasound transducers (CMUTs) technology into ICE‐guided catheter ablation techniques [11], taking advantage of CMUT's properties of better integration and providing higher signal‐to‐noise ratios to obtain higher quality images.

## Conflicts of Interest

The authors declare no conflicts of interest.
